# Coral Reef Community Composition in the Context of Disturbance History on the Great Barrier Reef, Australia

**DOI:** 10.1371/journal.pone.0101204

**Published:** 2014-07-01

**Authors:** Nicholas A. J. Graham, Karen M. Chong-Seng, Cindy Huchery, Fraser A. Januchowski-Hartley, Kirsty L. Nash

**Affiliations:** 1 ARC Centre of Excellence for Coral Reef Studies, James Cook University, Townsville, Queensland, Australia; 2 Geography, College of Life and Environmental Sciences, University of Exeter, Exeter, United Kingdom; California Polytechnic State University, United States of America

## Abstract

Much research on coral reefs has documented differential declines in coral and associated organisms. In order to contextualise this general degradation, research on community composition is necessary in the context of varied disturbance histories and the biological processes and physical features thought to retard or promote recovery. We conducted a spatial assessment of coral reef communities across five reefs of the central Great Barrier Reef, Australia, with known disturbance histories, and assessed patterns of coral cover and community composition related to a range of other variables thought to be important for reef dynamics. Two of the reefs had not been extensively disturbed for at least 15 years prior to the surveys. Three of the reefs had been severely impacted by crown-of-thorns starfish outbreaks and coral bleaching approximately a decade before the surveys, from which only one of them was showing signs of recovery based on independent surveys. We incorporated wave exposure (sheltered and exposed) and reef zone (slope, crest and flat) into our design, providing a comprehensive assessment of the spatial patterns in community composition on these reefs. Categorising corals into life history groupings, we document major coral community differences in the unrecovered reefs, compared to the composition and covers found on the undisturbed reefs. The recovered reef, despite having similar coral cover, had a different community composition from the undisturbed reefs, which may indicate slow successional processes, or a different natural community dominance pattern due to hydrology and other oceanographic factors. The variables that best correlated with patterns in the coral community among sites included the density of juvenile corals, herbivore fish biomass, fish species richness and the cover of macroalgae. Given increasing impacts to the Great Barrier Reef, efforts to mitigate local stressors will be imperative to encouraging coral communities to persist into the future.

## Introduction

The last two decades of coral reef research have seen a proliferation of publications charting the declining condition of coral reefs. These include reports of reductions in coral cover across entire regions or oceans [1], [2], [3], changes in coral community composition [4], [5], [6], [7], and associated losses in coral reef fish diversity and abundance [8], [9]. One way to better understand these changes is through research on community composition of reefs with differing disturbance history, which should also quantify ecological processes and physical features that may be important for reef condition [10]. Coral reef communities are known to vary with exposure to wave energy and the ‘zone’ of the reef on which they occur, such as the flat, crest or slope [11], [12], [13]. These differences are likely driven by variability in a range of natural factors, such as water movement, light penetration and temperature. However, whether these physical factors interact with disturbances to affect coral communities is poorly understood. Furthermore, the structural complexity of the reef habitat can differ greatly by reef zone and exposure, and may display varying vulnerability to structural collapse following severe disturbance [14], [15], [16]. A high level of structural complexity is fundamental to the persistence of a range of other organisms and ecological processes on coral reefs [reviewed by 17]. Therefore, varying levels of structural complexity may influence coral communities in the context of disturbance history, likely through indirect pathways. The majority of studies assessing coral condition in the context of disturbance and recovery have focussed on only one reef zone or level of exposure and many key variables, such as various ecological processes or physical attributes, are often not quantified [18].

Coral cover, when evaluated in isolation, is a fairly crude measure of coral reef condition, and may mask other changes in ecological processes or species composition [19], [20]. Indeed, many disturbances on coral reefs are non-random, with species-specific susceptibility leading to disproportionate declines in some taxa, resulting in shifts in the relative composition of species [4], [21], [22], [23], [24]. Importantly, these compositional shifts can persist through time [5], and may even lead to different species dominating a recovered reef [6], [7]. Although assessing coral species or genera composition is often informative, the extremely high diversity of coral reefs (the Great Barrier Reef, Australia has >450 known species of coral, for example) has led to various attempts to group species into ecologically meaningful categories. Categorising corals by growth morphology is one common way to group species [25]. For example, Wilson et al. [26] assessed recovery of corals in the inner Seychelles islands based on coral growth morphologies, and identified a shift from branching to encrusting forms through time. The use of coral trait information, such as growth rate, reproductive mode and growth form, has recently been used to identify four dominant life history strategies of corals that are globally consistent [27]. Importantly, these life history strategies (competitive, weedy, generalist and stress-tolerant) should reflect how groups of corals both respond to and recover from disturbances. For example, in a 20 year assessment of Kenyan reefs, stress-tolerant and weedy species varied least in response to a combination of fishing and bleaching disturbances, whereas competitive species became a much smaller component of the assemblage [28].

The Great Barrier Reef (GBR), Australia, is not immune to the general degradation of reef condition reported for many other reef regions of the world. Indeed, a series of large scale and influential publications have documented the declining coral cover and condition of the GBR, particularly in the mid to southern sections and in-shore habitats [19], . This is of major concern as the GBR is one of the largest reef systems in the world, supports an annual revenue of ∼AU$5.5 billion dollars through tourism and fisheries, provides a wide range of other ecosystem goods and services, and is a UNESCO World Heritage Area [32], [33]. Aside from the chronic impact of declining water quality following European clearance and farming of adjacent catchments [34], the Great Barrier Reef has experienced major large scale disturbances to coral cover in the form of predatory crown-of-thorns starfish (COTS) outbreaks, tropical cyclones, coral bleaching events and coral disease [31]. Of these, COTS outbreaks and cyclones are thought to have led to the most significant declines in live coral cover [35]. Despite these substantial disturbances, the Great Barrier Reef should be a very resilient system, capable of recovery. It is extremely large (∼3000 individual reefs, spanning 2300 kilometres), the reefs are relatively well connected [36], there is substantial deep water coral habitat [37], it is adjacent to a sparsely populated, affluent human population with relatively low reliance on fish protein [38], and good fisheries management and governance structures are in place [39]. With this high potential for recovery, it is important to assess the composition of reef communities, and assess key ecological processes that may correlate with these compositions, on reefs that have been exposed to varying disturbance histories.

Here we assessed the spatial community composition of five mid-shelf reefs of the Great Barrier Reef with known recent disturbance histories. Two of the reefs had relatively undisturbed communities for at least 15 years prior to the study, whereas coral cover on three of the reefs had been severely impacted by COTS and coral bleaching approximately 7–10 years prior to our surveys. Of these, only one of the reefs appeared to be recovering well, based on independent surveys. We assessed reef condition on these five reefs including data on coral cover, coral composition, coral recruit density, reef structural complexity and fish assemblages. For each reef we surveyed three reef zones (slope, crest and flat), at each of three sites on the wave exposed side of the reefs and at three sites on the sheltered side of the reefs. The extensive data we collected allowed a spatial assessment of the community composition of these reefs, in the context of known disturbance histories. Specifically we aimed to answer the following research questions: 1) How does coral cover, composition and structural complexity vary among reef zones and exposure levels on degraded or recently recovered reefs compared to reefs subject to little recent disturbance? 2) Which ecological predictor variables best correlate with coral compositional differences among these reefs?

## Methods

### Study sites

**Figure 1:**
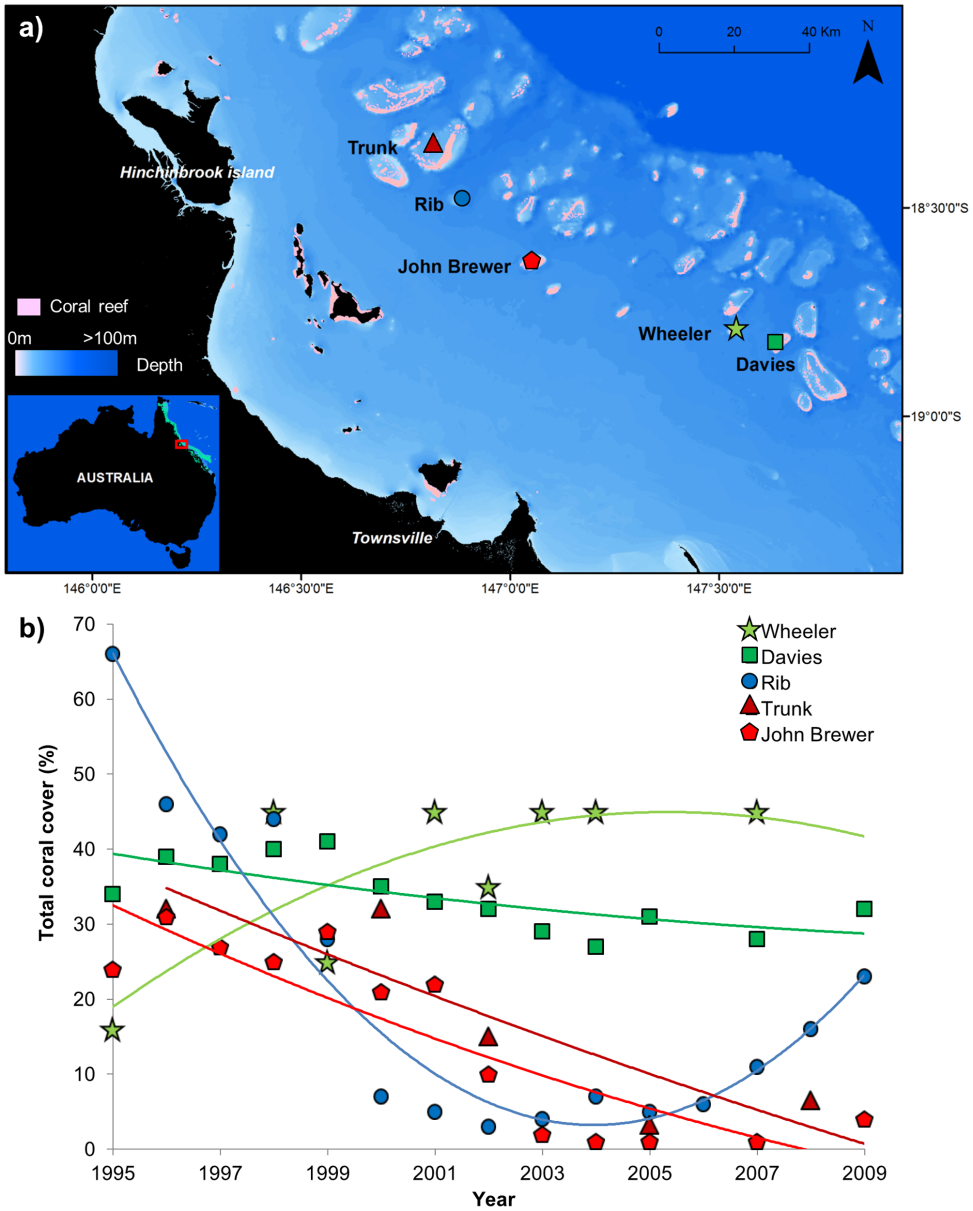
Location of studied reefs and historical patterns of coral cover through time. a) Map of the surveyed reefs. The green dots are the undisturbed reefs, the red dots are the disturbed reefs that did not recover, and the blue dot is the disturbed reef that recovered. b) Coral cover (%) estimates from the 5 reefs from 1995 to 2009 providing the disturbance history context for the spatial data collected in this study. Data for Trunk reef are from Pratchett et al. [40], and for Wheeler, Davies, Rib and John Brewer reefs are from the AIMS Long Term Monitoring Program [41].

We surveyed five mid-shelf reefs on the central section of the Great Barrier Reef near the city of Townsville (Fig. 1a). These reefs were chosen based on prior knowledge of their medium-term disturbance history [40], [41]. Wheeler and Davies reefs had been relatively undisturbed in the 15 years prior to the current surveys. Conversely, coral cover on Rib, Trunk and John Brewer reefs was severely impacted by a COTS outbreak between ∼1999–2003 and a coral bleaching event in 2001–02 [40], [41], leading to over 80% mortality of live coral, down to <5% absolute cover (Fig. 1b). Importantly, there was also variability in recovery rates; Rib reef appeared to be recovering its coral cover fairly rapidly, whereas Trunk and John Brewer reefs were showing much slower recovery [40], [41]. These differences in disturbance history and recovery rates provided a suitable design for a spatial comparison of differences in community composition and potential correlates of these differences. Aside from disturbance history, all other variables were held as constant as possible. For example, substantial variation in community composition of coral reefs of the Great Barrier Reef is well documented both across the shelf (inner-mid and outer shelf reefs) and with latitude along the length of the reef [11], [31], [42]. For that reason, all of the reefs we chose are in the same reef shelf position (mid-shelf) and of a restricted latitudinal range. Despite this, there is still potential for differences in hydrodynamic regimes and geographic features among reefs, which are hard to control for.

Wheeler reef is in a no-take ‘green’ zone of the GBR, whereas the other four reefs are in ‘yellow’ and ‘blue’ zones where fishing activities are allowed. The effects of the zoning on fish assemblages of the GBR are generally related to a small suite of target predatory species [43], with some indirect effects on fish prey [44]. Importantly, herbivorous fish species, which are thought to be important for reef recovery dynamics [29], are not commonly targeted by fisheries on the GBR. There is some evidence of reduced impacts of COTS in green zones of the GBR [45], with assessments of coral cover between no-take and fished reefs soon after an outbreak showing higher cover in no-take zones [32]. However, other work has shown no difference in coral cover between no-take and fished zones following the effects of various disturbances [46].

We examined the influence of wave exposure and reef zone, surveying six sites at each of the five reefs between November 2010 and January 2011. The predominant wave energy influencing reefs of the GBR comes from the south-east, particularly during the southern hemisphere winter trade wind months. Therefore, to capture any influence of exposure, we surveyed three sites on the south-east facing, wave exposed side of the reefs, and three sites on the north-east facing, wave sheltered side of the reefs. At each site, we collected data from 4 replicate transects in three different zones (slope, crest and flat). Surveys on the reef slope were between 7–9 m depth, parallel to the reef crest. The reef crest was well defined on all sites and survey depth was generally between 2–3 m depth. The reef flat communities were surveyed approximately 100 m back from, and parallel to, the reef crest.

All fieldwork was observational, non-extractive, data collection, and was conducted under research permit number G10/33239.1, issued by the Great Barrier Reef Marine Park Authority.

### Assessment of benthic and fish communities

We surveyed the benthic and fish communities along four 50 m replicate transects within each zone of each site. This resulted in a rich dataset of 72 transects per reef and 360 across all five reefs. The benthic composition was surveyed using the point intercept method, where the substrate directly below the transect tape was surveyed every 50 cm along each transect tape. Categories included sand, rubble, pavement, algae, hard and soft coral, and other benthic invertebrates (e.g. sponges). Hard corals were surveyed to the genus level and growth form noted, whereas algae were surveyed to broad functional classifications, such as turf, crustose coralline algae (CCA) and fleshy macroalgae. The density of coral recruits (colonies <1 cm diameter) and juvenile corals (colonies between 1 and 5 cm diameter) was quantified in a 10 m by 1 m belt transect at the start of each 50 m transect tape. The structural complexity of the reef at each transect was estimated visually on a six point scale, following Polunin & Roberts [47]. The scale ranges from no vertical relief, to exceptionally complex habitats with numerous caves and overhangs. This measure of structural complexity captures a broad picture of the structure, has been shown to correlate well with a range of other structural complexity measurement techniques, and to correlate with the density and diversity of coral reef fish assemblages [48].

All diurnally active, non-cryptic, reef associated fish species >8 cm total length (TL) were surveyed. Larger more mobile species were counted as transects were laid in a 5 m wide belt, and small territorial species (mostly pomacentrids and some labrids) were surveyed on the return pass along the transect in a 2 m wide belt. Individual fish were identified to species, their abundance counted and their size estimated to the nearest centimetre (TL). Length estimation was calibrated at the start of each day's diving by estimating the length of a random selection of PVC pipes. A total of 261 species of fish from 27 families were recorded in the surveys. Fish survey data were converted to biomass using published length-weight relationships [49], [50]. Species were assigned to feeding groups (corallivores, herbivores, invertivores, mixed diet, piscivores) based on the literature and dietary information [50].

### Data analyses

Broad differences in the percent cover of live coral, structural complexity and the coral genus richness per reef were initially assessed using one-way ANOVAs and Tukey's post-hoc tests to identify which reefs were driving any differences. A more detailed assessment of the variation in these three response variables was assessed using a hierarchical model, with wave exposure (exposed and sheltered) and zone (slope, crest and flat) as fixed factors that also include their interaction, and reef as a random effect. Normality of the residuals and homogeneity of variances were assessed by reviewing plots of residuals against fitted values and Q-Q plots, and all assumptions of the test were met.

We classified our coral genera/growth form data into the four life history strategies defined by Darling et al. [27] (Table S1). We used the direct genera classifications presented in Darling et al. [27] where available, and the description of the categories for the remaining coral genera. Competitive corals are described as large, branching and plating species that grow quickly, occur at shallow depths and reproduce by broadcast spawning. Weedy corals are described as species that can reproduce by brooding and have smaller colony sizes. Stress-tolerant corals are slow-growing species that reproduce by broadcast spawning and have primarily massive/domed morphologies, large corallites and high fecundity. Generalist corals include an assortment of species that show some overlap with the competitive, weedy and stress-tolerant life histories. We included a fifth category, other, which included corals that we could not classify into one of the four life history groups with genus/growth form data.

We assessed differences in square root transformed coral composition (based on the above life history categorisation) among the reefs, zones and exposure levels using non-metric multidimensional scaling (MDS) on a Bray-Curtis similarity matrix. We used a three-way PERMANOVA, with interactions (maximum permutations  = 9999), to test whether the coral group composition differed significantly among sites experiencing different levels of 3 factors: reef, zone (slope, crest, flat) and exposure (exposed, sheltered). We ran the same analyses using the original coral genera/growth form data as a comparison to the results of the life history categorisation of the corals.

We assessed broad differences among the reefs in a range of key variables that are known to have the potential to influence coral community dynamics using one-way ANOVAs. These variables included total fish biomass (kg/ha), herbivore biomass (kg/ha), number of coral juveniles (1–5 cm), number of coral recruits (<1 cm), macroalgae cover (%), fish species richness per sites, and CCA cover (%). We used Tukey's post-hoc tests to identify which reefs drove any identified differences. Assumptions of the test were assessed by reviewing plots of residuals against fitted values and Q-Q plots. To assess which combination of these predictor variables was best correlated with the site level patterns in the coral communities, we first normalised the predictor variables listed above to put them on the same scale, and log transformed them to improve the spread of the data. We then used the rank-correlation BEST BIO-ENV routine to assess which combination of the predictor variables best correlated with the patterns in the coral life history community data. The technique computes rank-correlations for all possible combinations of predictor variables, and converges on the combination with the strongest relationship with the dependent community composition dataset [51]. The significance of the relationship between the predictor variables and the coral life history composition data was assessed using a permutation test, that randomly reassigns sample lables multiple times to create a null distribution of potential correlation coefficients to compare the actual value to. This analysis was performed on the entire dataset, and for the three disturbance categories (undisturbed, recovered, unrecovered) of reefs separately to see if the recovered reef had a different set of predictor variables from the unrecovered reefs. Because the BEST BIO-ENV routine does not provide information on the direction of influence of predictor variables, we also performed a redundancy analysis (RDA) to examine the directions of the relationships between the predictor variable data matrix and the coral community data matrix.

## Results

**Figure 2:**
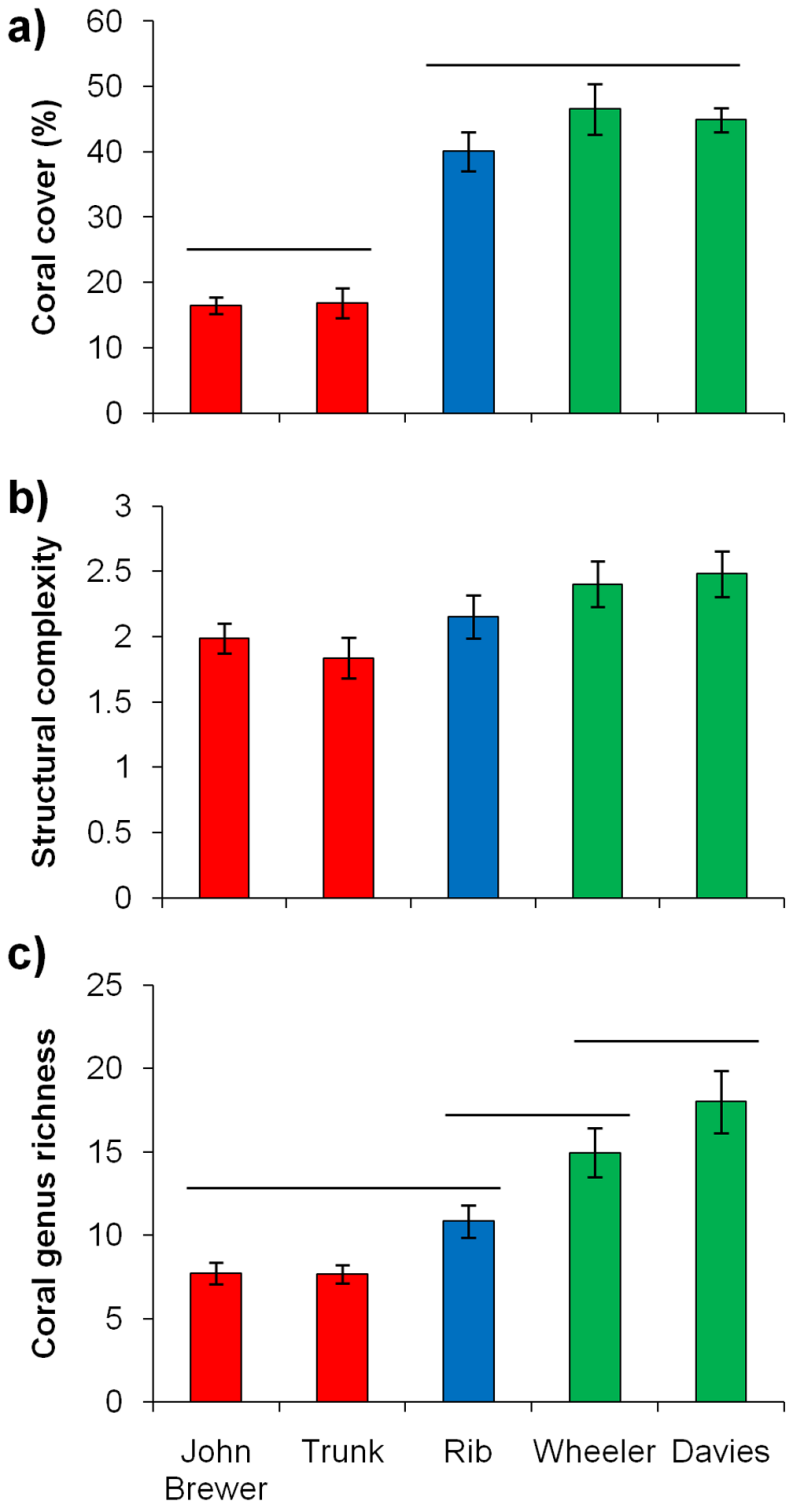
Variation in: a) coral cover (%), b) structural complexity, and c) coral genus richness among reefs. Red bars are unrecovered reefs, blue bar is the recovered reef and green bars are undisturbed reefs. Bars represent means per site ±standard error. Horizontal lines represent homogeneous subsets from post hoc comparisons using the Tukey test.

Assessing broad differences at the reef scale identified significant differences in coral cover (F_4,85_ = 32.89, p<0.001), structural complexity (F_4,85_ = 2.98, p = 0.024), and coral genus richness (F_4,85_ = 14.19, p<0.001) (Fig. 2). Specifically, the undisturbed reefs, which were similar to each other for all three variables, generally exhibited significantly higher coral cover, structural complexity and coral genera richness than the two unrecovered reefs (Trunk and John Brewer; Fig. 2). In comparison, the recovered reef (Rib) had similar coral cover and structural complexity to the undisturbed reefs (Wheeler and Davies), but was only similar to one of the undisturbed reefs (Wheeler) in terms of coral genera richness. The total coral genus richness recorded at the reef level was 39 and 43 for Wheeler and Davies reefs, 36 for Rib reef, and 27 and 30 for John Brewer and Trunk reefs.

**Figure 3:**
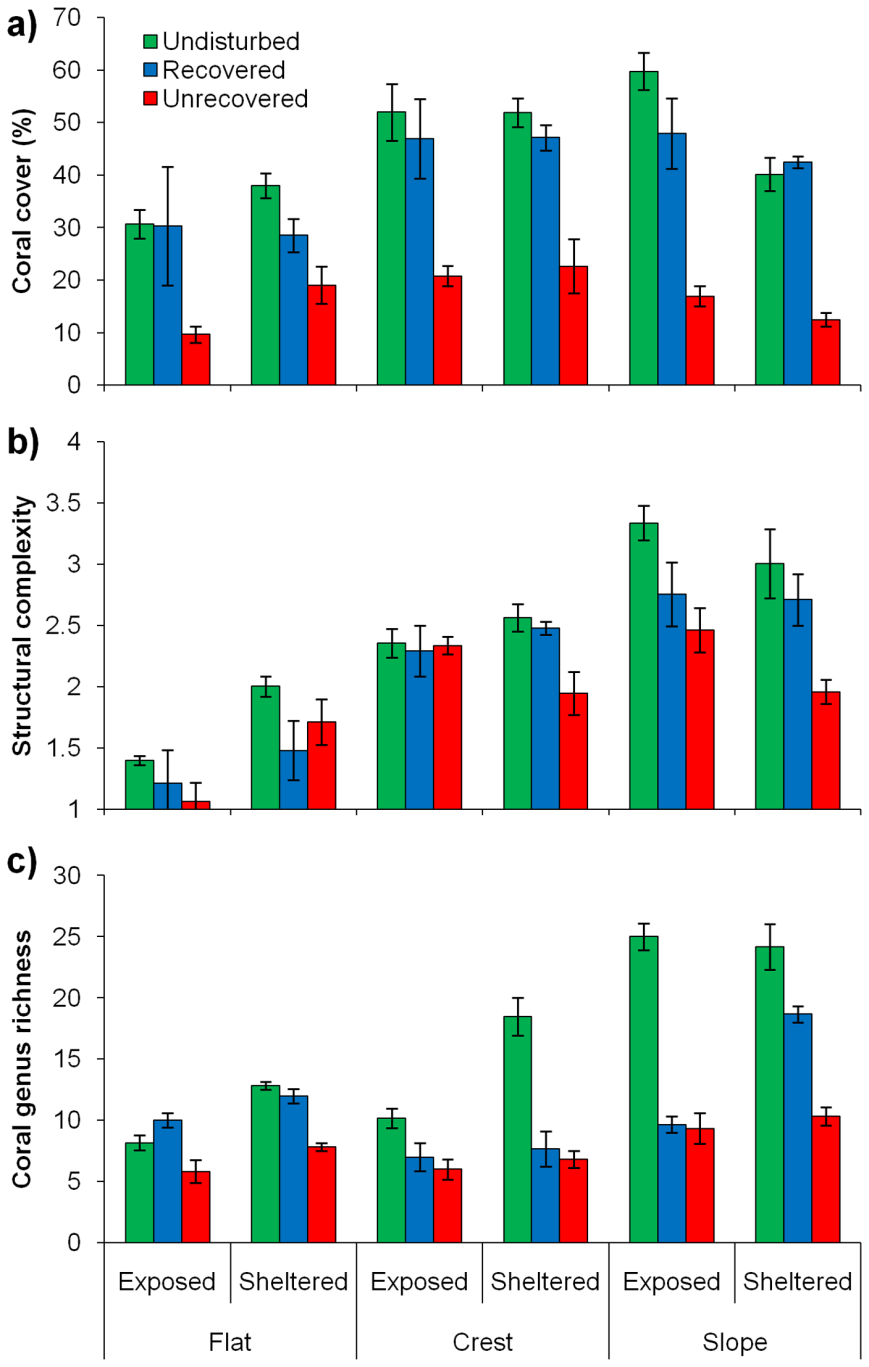
Influence of zone and exposure on coral cover, richness and structural complexity among disturbance groupings. Variation in: a) coral cover (%), b) structural complexity, and c) coral genus richness among disturbance category, reef zones and wave exposure levels. Bars represent means per site ± standard error.

Assessing coral cover, structural complexity and coral genus richness among zones and wave exposure treatments exposed more of the variation in the data (Fig. 3). The random factor, reef, separated out the reefs similar to the one-way ANOVA above with respect to disturbance history (Table S2). There was a significant effect on coral cover among reef zones and for the interaction among zones and exposure (Table 1). Coral cover was generally higher on sites in reef crest and slope habitats, compared to reef flat habitats (Table 1, Fig. 3a). Coral cover was 2 to 3 times lower on the unrecovered reefs, than the undisturbed and recovered reefs, regardless of zone or exposure level, indicating that all of these environments are vulnerable to coral loss through COTS and/or bleaching (Fig. 3a). For the recovered reef, live coral cover was comparable to the undisturbed reefs across all zones and exposures, except for wave sheltered reef flat environments and exposed reef slopes, where it was lower (Fig. 3a).

**Table 1:** Sources of variation in coral cover, structural complexity and coral genus richness among zones and exposure.

	Value	Std.Error	Df	t-value	p-value	Sig
**a) Hard coral cover**						
(Intercept)	38.99	6.59	80	5.920	0.0000	***
Flat	−16.78	3.29	80	−5.105	0.0000	***
Slope	1.05	3.29	80	0.319	0.7509	
Sheltered	0.07	3.29	80	0.020	0.9839	
**b) Structural complexity**						
(Intercept)	2.34	0.15	80	15.959	0.0000	***
Flat	−1.11	0.14	80	−7.660	0.0000	***
Slope	0.53	0.14	80	3.687	0.0004	***
Sheltered	−0.04	0.14	80	−0.249	0.8043	
**c) Coral genus richness**						
(Intercept)	7.87	2.06	80	3.812	0.0003	***
Flat	−0.27	1.30	80	−0.205	0.8378	
Slope	7.80	1.30	80	6.006	0.0000	***
Sheltered	3.80	1.30	80	2.926	0.0045	**

Hierarchical model results assessing: exposure (exposed, sheltered); and zone (flat, crest, slope) and their interaction, with reef as a random factor for a) coral cover (%), b) structural complexity, and c) coral genus richness. Significance levels: ***<0.001, **<0.01, *<0.05. Crest and exposed are the intercept. The interaction of zone and exposure had a significant impact on hard coral cover and structural complexity.

For structural complexity there were also significant effects of reef zone and the interactions between exposure and zone (Table 1). Overall structural complexity was substantially lower on the reef flat habitats than reef crest and slope habitats, and this was particularly the case for exposed locations (Fig. 3b). Differences in structural complexity among sites associated with disturbance were restricted to the reef slope habitats and the crest habitats on sheltered sides of reefs (Fig. 3b). The structural complexity of the recovered reef generally fell somewhere between that of the undisturbed and unrecovered reefs (Table S2, Fig. 3b).

There were significant differences for coral genus richness values among sites, driven by wave exposure and reef zone (Table 1). Coral genus richness on unrecovered reefs was lower than on undisturbed reefs at all reef zones and exposure levels, and lower than on recovered reefs on the reef flat and sheltered slope sites (Table S2, Fig. 3c). The recovered reef had similar levels of coral genus richness as the undisturbed reefs for the reef flat sites, but lower values for the reef crest and slope habitats, although in the sheltered slope environment it was approaching the levels of the undisturbed reefs (Fig. 3c). For the undisturbed reefs, richness was highest on the reef slope and sheltered crest habitats and lowest on the reef flat and exposed crest habitats (Fig. 3c).

**Figure 4:**
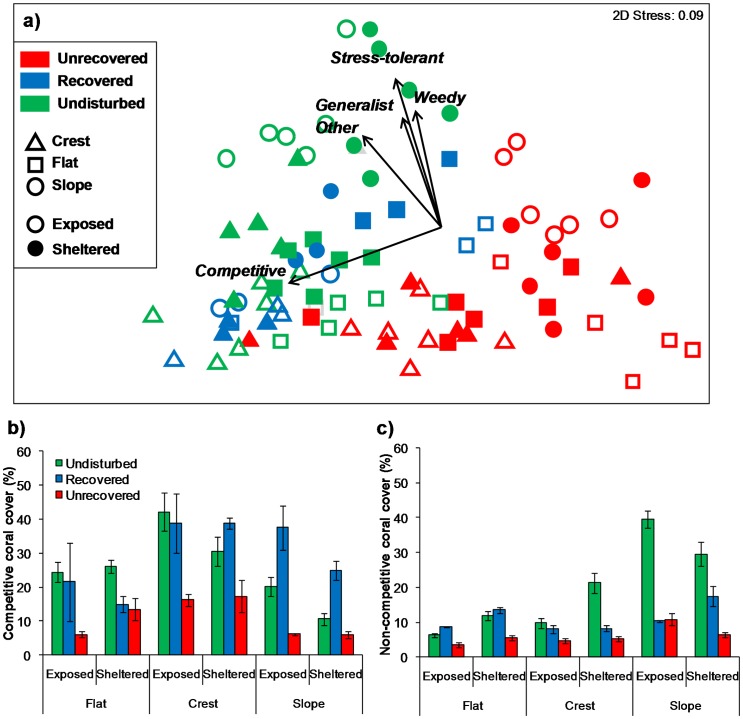
Differences in coral community composition by life history strategy with disturbance, zone and exposure. a) Non-metric multidimensional scaling analysis of coral group cover (%) based on life history categorisation. Colour and shape of symbols represent disturbance category, reef zone and wave exposure. Vectors represent the relative contribution of the coral groups to the observed variation among sites. b) Competitive coral cover (%) among disturbance category, reef zone and wave exposure. c) Non-competitive (weedy, stress-tolerant, generalist and other) coral cover (%) among disturbance category, reef zone and wave exposure. Bars represent means per site ± standard error.

The coral communities based on life history groups highlight substantial differences in composition among reefs, exposure and zone (Fig. 4a, Table 2). Pairwise tests among the reefs grouped them by disturbance history; the undisturbed reefs (Wheeler and Davies) were similar, as were the two unrecovered reefs (John Brewer and Trunk), while the recovered reef (Rib) was different to all other reefs except Wheeler (Table S3). The sites at unrecovered reefs all cluster away from the other reef sites, with the lowest values of cover for all coral life history strategies. The undisturbed sites generally had a mix of coral life history strategies dominating, particularly for the reef slope habitats. Conversely, the recovered reef sites, particularly for reef slope and crest habitats, are skewed towards the competitive coral life history group, which likely helps explain why many of these sites have a lower genera richness than the undisturbed sites. Assessing the cover of these life history groups by treatment helps uncover these patterns in more detail. Competitive corals were responsible for the majority of cover on the recovered reef, particularly in the reef slope and crest habitats (Fig. 4b, Table S4 and S5). Indeed, in the slope habitats, this group of corals was far more dominant on the recovered reef than on the undisturbed reefs. Conversely, the other groups of corals (lumped into non-competitive here) had substantially higher cover on undisturbed reefs in the reef slope habitats and sheltered crests (Fig. 4c, Table S4 and S5). Much of this difference in non-competitive corals was driven by stress tolerant genera, with some generalist, weedy and unclassified (other) corals also contributing, particularly in exposed slope habitats (Table S4 and S5, Fig. S1). Although grouping corals into these life history categories helps to explain a complex community with relatively few groups, it is important to assess if very different results occur if raw coral genera data are used. Analysing the data in the same way using coral genera level data produced a very similar ordination, with the three disturbance categories separating out, and the branching and tabular *Acropora* corals accounting for of the cover in the recovered reef category (Fig. S2).

**Table 2:** Results of PERMANOVA test on coral life history strategy data.

Source	df	SS	MS	Pseudo-F	P(perm)
**reef**	4	33242	8310.4	27.267	0.001
**expo**	1	1232	1232	4.0422	0.011
**zone**	2	17552	8775.8	28.795	0.001
reef*expo	4	3539.5	884.88	2.9034	0.001
reef*zone	8	13766	1720.7	5.6458	0.001
expo*zone	2	2353.4	1176.7	3.8609	0.003
reef*expo*zone	8	3097.5	387.19	1.2704	0.18
**Residuals**	60	18286	304.77		
**Total**	89	93068			

Factors: reef (Wheeler, Davies, Rib, Trunk, John Brewer); expo  =  exposure (exposed, sheltered); and zone (flat, crest, slope).

Assessing variables that are thought to influence reef dynamics at a broad reef scale highlighted significant differences in fish biomass, herbivore biomass, the number of juvenile corals and the cover of macroalgae (Table 3). Fish biomass and herbivore biomass were both highest on Wheeler reef. Of particular note on the only disturbed reef to have recovered in our study (Rib reef), the number of juvenile corals was the greatest and the cover of macroalgae was the lowest of all the reefs studied (Table 3). The BEST BIO-ENV analyses showed that considerable amounts of the variation in coral composition (based on cover of different life history groups) were associated with the predictor variables (Table 4). For all reefs combined, the best set of predictor variables associated with patterns in the coral community were herbivore biomass (most associated with recovered and undisturbed reefs and competitive coral cover on the RDA), fish species richness (most associated with other types of corals on undisturbed reefs on the RDA), and the cover of macroalgae, which was most associated with unrecovered reefs (Table 4, Fig. S3). In the subset of sites on undisturbed reefs, macroalgae cover alone had a correlation of 0.52 with the coral community composition. For the reef that recovered, fish biomass, coral juveniles and fish species richness produced a correlation of 0.60 with the coral community. For unrecovered reefs, a correlation of 0.29 was found, including the variables herbivore biomass, the density of coral juveniles and fish species richness (Table 4). Interestingly, herbivore biomass was negatively correlated with coral juvenile density (*r_s_* = −0.28, *p*<0.01), but positively correlated with total hard coral cover (*r_s_* = 0.41, *p*<0.001).

**Table 3:** Variation in total fish biomass, herbivore biomass, number of coral juveniles, number of coral recruits, macroalgae cover, fish species richness, and CCA cover among reefs.

	Undisturbed		Recovered	Unrecovered		
	Wheeler	Davies	Rib	John Brewer	Trunk	Significant differences (p-value<0.05)
**Fish biomass (kg/ha)**	993.6 (±123.4)	771.8 (±97.2)	625.0 (±77.1)	595.7 (±79.5)	478.7 (±62.4)	Wheeler > John Brewer, Rib, Trunk
**Herbivore biomass (kg/ha)**	566.1 (±47.0)	453.4 (±57.3)	387.4 (±47.2)	343.5 (±53.6)	265.5 (±31.5)	Wheeler > John Brewer, Trunk
**Number of coral juveniles (<5 cm) per site**	46.1 (±4.6)	63.1 (±4.6)	96.6 (±9.1)	60.8 (±6.0)	59.1 (±7.2)	Rib > Davies, John Brewer, Trunk, Wheeler
**Number of coral recruits (<1 cm) per site**	1.7 (±0.3)	2.0 (±0.4)	1.6 (±0.3)	1.2 (±0.3)	1.6 (±0.4)	-
**Macroalgae cover (%)**	5.3 (±1.4)	7.8 (±1.7)	0.7 (±0.2)	9.9 (±1.4)	12.7 (±2.5)	Rib < Davies, John Brewer, Trunk, Wheeler
**Fish species richness per site**	51.3 (±2.3)	50.4 (±2.3)	55.5 (±4.0)	52.2 (±3.2)	47.7 (±2.7)	-
**CCA cover (%)**	9.7 (±1.4)	14.9 (±1.6)	12.2 (±1.0)	12.0 (±1.3)	10.7 (±1.6)	-

The values are means per site ± standard error. The last column highlights significant differences among the reefs.

**Table 4:** BEST BIO-ENV results for all reefs combined and the three disturbance categories separately.

Sites	Rho	p-value	Variables selected
**All**	0.315	<0.001	herbivore biomass, fish species richness, macroalgae cover
**Undisturbed**	0.515	<0.001	macroalgae cover
**Recovered**	0.599	<0.001	fish biomass, coral juveniles, fish species richness
**Unrecovered**	0.288	<0.001	herbivore biomass, coral juveniles, fish species richness

Potential predictor variables included were fish biomass, herbivore biomass, number of coral juveniles, number of coral recruits, macroalgae cover, fish species richness and CCA cover. Rho value is the spearman rank correlation, or amount of variation in coral composition/cover associated with the selected predictor variables. P-value is calculated from a permutation test.

## Discussion

We found substantial differences among reefs, zones and exposure for coral cover, structural complexity and the richness of coral genera. Although the recovered reef appeared to be reaching coral cover levels of the undisturbed reefs, the coral composition differed substantially, particularly in reef slope and crest habitats. This may support previous studies showing long-term shifts in coral composition following disturbances and recovery [5], [6], [7], or may represent a different natural climax community on this reef. The composition and cover of unrecovered reefs was quite depauperate, even ∼1 decade post-disturbance. Interestingly, the ecological predictor variables we collected were quite strongly correlated with these coral composition patterns, suggesting predictable relationships that may be of use for management.

Rib reef was the only one of our three disturbed reefs that had demonstrated substantial recovery of live coral cover (inferred from the AIMS long term monitoring data and the status of the reef in our spatial surveys). The recovery time period (∼1 decade) is similar to other published examples of rapid reef recovery following severe disturbance [52], [53], [54], [55]. The broad reef attributes unique to Rib reef were: a higher density of juvenile corals than at any other reef, and lower cover of macroalgae. Juvenile coral survivorship has been shown to be key to reef recovery dynamics [55] and low levels of macroalgae cover can also greatly increase the rate of recovery of hard coral cover [26]. Rib reef has previously been shown to have high coral recruitment (especially *Acropora*) and low coral recruit mortality [56]. Rib reef sits within the Palm Passage, a major zone of GBR water inflow from the Coral Sea [57], and it has been suggested that high coral recruitment rates could be explained by water exchange between the outer shelf and Rib Reef along the Palm Passage [58]. Furthermore, high recruit survivorship and potentially retarded macroalgae growth may be aided by lower levels of terrestrial influence, due to the separation of Rib reef from the inshore environment by the Palm islands [59]. Such geological and hydrological explanations for differing reef dynamics are poorly understood, but may be important variables to understand and incorporate into management planning.

The substantial compositional differences observed in our data beg the question as to whether these are natural differences, slow successional processes that will converge through time, or if these differences in composition are likely to be permanent. The composition of corals at Rib reef reflect recovery back to compositions seen on that reef in the past. Data from Rib reef in the 1980's and 1990's indicate a dominance of (plating) *Acropora* corals [41], [60], during a time when the reef had been recovering from recurrent COTS impacts. Interestingly, dominance by plating *Acropora* corals was also apparent for John Brewer reef in the 1990's [41], yet this reef had low coral cover, including plating *Acropora* in the current spatial surveys. Succession theory suggests that while biomass or cover may recover rapidly, it can take a lot longer for species diversity and community composition metrics to reach similar levels as undisturbed communities [61], [62], [63]. A recent temporal study of 6 reefs on the Great Barrier Reef indicted that reefs recovered coral cover faster than reassembling composition, and some reefs appeared unlikely to reassemble their pre-disturbance compositions [64]. The authors suggested that many reefs are unlikely to return to pre-disturbance compositions if disturbance events become too frequent. Indeed, coral genera responses to many disturbances are non-random, with some genera being considerably more susceptible than others [4], [65], [66], and non-random re-shuffling of coral composition may lead to longer-term novel ecosystem configurations that are unlikely to continue to recover to pre-disturbance configurations [67]. It is not possible to distinguish among these various possibilities for differing compositions with snapshot spatial data, such as that presented here. However, this is clearly a topic in need of substantial research attention given the increasing frequency of disturbances impacting coral reefs.

Assigning coral genera and growth forms into life history groupings provided a mechanism to simplify the information presented, and highlights some interesting patterns in the data. Most of the cover recorded for Rib reef was associated with competitive corals, which are principally *Acropora* species. This genus is known to be fast growing, and has been shown to underpin rapid recovery trajectories [54], [55]. However, this finding contrasts with the trajectory of the competitive life history group through a major disturbance event in Kenya, where recovery rates were slow, particularly in heavily fished sites [28]. In our data, the cover of competitive corals on reef slope habitats of the recovered reef exceeded the cover recorded on the undisturbed reef, which may reflect the space available for rapid colonisation and growth following a large disturbance. Indeed, Graham et al. [18] found that coral recovery rates were fastest when coral cover had been reduced the most, and hence more space was available. The undisturbed reefs had higher cover of the non-competitive life history strategy coral groups, reflective of the higher diversity on these reefs. Interestingly, stress-tolerant groups of corals had substantially lower cover on both the unrecovered reefs and recovered reef, than the undisturbed reefs, aside for on reef flat habitats. Darling et al. [28] found that the stress-tolerant group of corals fared well in response to coral bleaching and fishing impacts in Kenya. COTS are known to be fairly selective in their feeding on different genera of corals, and some of the corals they actively select (such as *Favites* and *Montipora*) are in the stress-tolerant group [22], indicating that this type of disturbance has the potential to greatly reduce cover of even the corals that are thought to be tolerant to other stressors. Similar to the findings of Darling et al. [28], we found that weedy coral taxa were fairly well represented on the recovered reef, for example in the exposed crest and sheltered slope habitats. Repeating the analysis with the coral genera community data produced a very similar ordination, indicating that the life history groupings provided a useful representation of the community response to disturbance. As mentioned above, it is hard to say whether our reefs with different recent disturbance histories would have had similar coral compositions prior to disturbance. Although the size, latitude and shelf position of the reefs was held as consistent as possible in our design, other factors such as hydrological and geographic variables may be important.

Our BEST BIO-ENV and RDA results highlighted strong associations between sub-sets of our predictor variables and patterns in coral community structure in our study. While the predictor variables included were selected as they are known to be potential drivers of reef community dynamics, it should be noted that our predictor variables could be both causes and consequences of the observed differences in coral communities. When all the reefs were included, the most important variables were herbivore biomass and fish species richness, which were higher on undisturbed and recovered reefs, and macroalgae cover, which was generally higher on unrecovered reefs. Higher herbivore biomass is known to negatively influence macroalgae cover [68], [69], and grazing should be beneficial to coral recruitment and survival [70]. Similarly, fish species richness, can be an important determinant of ecosystem function on coral reefs [71] and was found to be one of the key factors influencing why some GBR reefs enter a phase shift to algae and fail to recover, whereas others do not [42], [72]. However, fish species richness is also responsive to changes to benthic condition [8], [9], [15], making it hard to tease out cause and effect in these data. When solely assessing undisturbed reefs, macroalgae cover alone was strongly correlated with coral community patterns. Competition between macroalgae and corals is known to influence benthic community dynamics on coral reefs [73], and in the absence of disturbance may be one of the dominant processes shaping community composition. Interestingly, the strongest correlation was for the reef that had recovered it's coral cover, with fish biomass, coral juvenile density and fish species richness, all important variables. Fish biomass has recently been shown to mediate a range of processes and ecosystem state variables in coral reefs, with very low levels of biomass expected to retard the ability of reefs to recover [20]. The fish biomass recorded for the GBR here is relatively high and above the levels at which many important ecological processes are thought to be lost [20]. The recovered reef (Rib) had the highest densities of juvenile corals, which has been shown to be important for reef recovery [55]. The weakest correlation was for the unrecovered reefs, with herbivore biomass, juvenile coral density and fish species richness selected as important variables.

The potential for an interaction between herbivore biomass and juvenile corals could be important for degraded reefs. Similar to Trapon et al. [74], we found a negative correlation between herbivore biomass and juvenile coral density across our study sites. However, we also found a positive correlation between herbivore biomass and adult coral cover. Similar to the Caribbean [75], the relationships between herbivore biomass, algal cover and coral survivorship appears to be complex in the Indo-Pacific region, although the beneficial effects of controlling algae are likely to be most important in influencing positive trends in live coral cover [26].

Reef zonation is well known to influence both coral cover and the composition of species present [11], and was responsible for a substantial amount of the variation in our data, whereas wave exposure was only responsible for a small amount of variation. The highest coral cover for the undisturbed and recovered reefs was on the reef slope and crest, with the sheltered slope environment appearing to have the lower cover. Reef flat environments had the lowest cover overall. Structural complexity and coral genera richness were generally highest on the slope, then the crest and lowest on the flat. The reef flat environment, which had the lowest level of coral cover, likely has high wave energy and water motion, factors that are known to influence coral growth form and species composition [76]. Structural complexity was collinear with live coral cover in our data, so could not be used as a predictor variable. Live coral does contribute directly to structural complexity, but complexity is also provided by the structure in the underlying matrix of the reef [77]. Structural complexity is known to be important to a range of ecological groups and processes on coral reefs [17], [78], and the reduced complexity observed on wave sheltered reef slope and crest sites of the unrecovered reefs may slow rates of eventual recovery on those reefs.

Shortly after the surveys reported here were conducted, cyclone Yasi, a very large category 5 storm passed over the study reefs and caused extensive damage to both coral cover and the structural complexity of the reefs [80]. Another category 5 cyclone (cyclone Hamish) had damaged a 500 km section of the southern GBR in 2009 [79]. Furthermore, a new COTS outbreak has been developing in the northern GBR [41]. The frequency of these acute impacts and the effects of coral bleaching and disease, are major concerns for the condition of the GBR, and the longer-term trajectories for coral cover indicate ongoing loss [29], [30], [31]. Many of these impacts, such as cyclones and bleaching, are difficult to manage locally, but policies to mitigate local threats should give the reefs the best chance possible of being resilient and bouncing back from increasing disturbance regimes [19], [80]. Fishing is already well managed on the GBR [81]. Improving water quality, including terrestrial pollutants, sediments and nutrients, should be a priority given evidence of inshore reef degradation and links between water quality and COTS outbreaks [82]. Furthermore, water quality can influence macroalgae cover [83] and coral recruitment and survivorship [84], two of the key variables we identified as related to patterns of coral community structure in this study.

## Supporting Information

Figure S1 **Cover of four coral life history categorisations at reef sites of different disturbance, zone and exposure.** a) Stress-tolerant, b) weedy, c) generalist, d) other coral cover (%) among disturbance category, reef zone and wave exposure. Bars represent means per site ± standard error.
(DOCX)

Figure S2 **Differences in coral community composition by genera with disturbance, zone and exposure.** Non-metric multidimensional scaling analysis of coral genera/growth form cover (%). Colour and shape of symbols represent disturbance category, reef zone and wave exposure. Vectors represent the relative contribution of selected coral genera/growth form to the observed variation among sites. Vectors represented have a minimum correlation of 0.6 with MDS1 or MDS2.
(DOCX)

Figure S3 **Relationships between predictor variables and coral community composition patterns.** Redundancy analysis of coral life history composition patterns on reef sites of different disturbance history, zone and wave exposure level, with the biological predictor data overlaid to demonstrate the direction of influence of the various variables.
(DOCX)

Table S1 **Classification of coral genera/growth forms into the coral life history groups defined in Darling et al. (2012).**
(DOCX)

Table S2 **Random factor “reef” results of the hierarchical models for a) hard coral cover, b) structural complexity and c) coral genus richness.** The intercepts and 95% confidence intervals (CI) of each reef and their corresponding disturbance are presented.
(DOCX)

Table S3 **Pairwise tests among reefs for PERMANOVA results on coral community composition data.**
(DOCX)

Table S4 **Hierarchical model results assessing exposure and zone and their interaction with reef as a random factor for: a) competitive, b) non competitive, c) stress-tolerant, d) weedy, e) generalist, f) other coral cover (%).**
(DOCX)

Table S5 **Random factor “reef” results of the hierarchical models for a) competitive, b) non competitive, c) stress tolerant, d) weedy, e) generalist, f) other coral cover (%).** The intercepts and 95% confidence intervals (CI) of each reef and their corresponding disturbance are presented.
(DOCX)
