# Supplementary material for: Coral Reef Community Composition in the Context of Disturbance History on the Great Barrier Reef, Australia
Source: PLoS One. 2014 Jul 1;9(7):e101204. doi: 10.1371/journal.pone.0101204 (PMC4077760; doi:10.1371/journal.pone.0101204)
Supplement: Figure S1 — Cover of four coral life history categorisations at reef sites of different disturbance, zone and exposure. a) Stress-tolerant, b) weedy, c) generalist, d) other coral cover (%) among disturbance category, reef zone and wave exposure. Bars represent means per site ± standard error. (DOCX) [file pone.0101204.s001.docx]

**Figure S1.** **Cover of four coral life history categorisations at reef sites of different disturbance, zone and exposure.**

a) Stress-tolerant, b) weedy, c) generalist, d) other coral cover (%) among disturbance category, reef zone and wave exposure. Bars represent means per site ± standard error.
