# Supplementary material for: Coral Reef Community Composition in the Context of Disturbance History on the Great Barrier Reef, Australia
Source: PLoS One. 2014 Jul 1;9(7):e101204. doi: 10.1371/journal.pone.0101204 (PMC4077760; doi:10.1371/journal.pone.0101204)
Supplement: Figure S2 — Differences in coral community composition by genera with disturbance, zone and exposure. Non-metric multidimensional scaling analysis of coral genera/growth form cover (%). Colour and shape of symbols represent disturbance category, reef zone and wave exposure. Vectors represent the relative contribution of selected coral genera/growth form to the observed variation among sites. Vectors represented have a minimum correlation of 0.6 with MDS1 or MDS2. (DOCX) [file pone.0101204.s002.docx]

**Figure S2. Differences in coral community composition by genera with disturbance, zone and exposure.**

Non-metric multidimensional scaling analysis of coral genera/growth form cover (%). Colour and shape of symbols represent disturbance category, reef zone and wave exposure. Vectors represent the relative contribution of selected coral genera/growth form to the observed variation among sites. Vectors represented have a minimum correlation of 0.6 with MDS1 or MDS2.
