# Supplementary material for: Coral Reef Community Composition in the Context of Disturbance History on the Great Barrier Reef, Australia
Source: PLoS One. 2014 Jul 1;9(7):e101204. doi: 10.1371/journal.pone.0101204 (PMC4077760; doi:10.1371/journal.pone.0101204)
Supplement: Figure S3 — Relationships between predictor variables and coral community composition patterns. Redundancy analysis of coral life history composition patterns on reef sites of different disturbance history, zone and wave exposure level, with the biological predictor data overlaid to demonstrate the direction of influence of the various variables. (DOCX) [file pone.0101204.s003.docx]

**Figure S3. Relationships between predictor variables and coral community composition patterns.**

Redundancy analysis of coral life history composition patterns on reef sites of different disturbance history, zone and wave exposure level, with the biological predictor data overlaid to demonstrate the direction of influence of the various variables.
