# Supplementary material for: Coral Reef Community Composition in the Context of Disturbance History on the Great Barrier Reef, Australia
Source: PLoS One. 2014 Jul 1;9(7):e101204. doi: 10.1371/journal.pone.0101204 (PMC4077760; doi:10.1371/journal.pone.0101204)
Supplement: Table S1 — Classification of coral genera/growth forms into the coral life history groups defined in Darling et al. (2012). (DOCX) [file pone.0101204.s004.docx]

**Table S1.** Classification of coral genera/growth forms into the coral life history groups defined in Darling et al. (2012).

| **Group** | **Coral genera/growth form** |
| --- | --- |
| **Competitive** | *Acropora* branching, *Acropora* submassive, *Acropora* tables, *Montipora* foliose |
| **Generalist** | *Echinopora* branching, *Echinopora* encrusting, *Echinopora* plating, *Hydnophora* plating, *Merulina*, *Montipora* foliose, *Mycedium*, *Pachyseris*, *Psammacora*, *Turbinaria* |
| **Weedy** | *Leptastrea*, *Porites* branching, *Seriatopora*, *Stylophora* |
| **Stress-tolerant** | *Acanthastrea*, *Alveopora*, *Astreopora*, *Caulastrea*, *Cyphastrea*, *Diploastrea*, *Echinophyllia*, *Favia* (plocoid), *Favites* (ceriod), *Fungiidae*, *Galaxea*, *Gardinoseris*, *Goniastrea*, *Hydnophora* branching, *Leptoria*, *Lobophyllia*, *Montastrea*, *Montipora* encrusting, *Oulophyllia*, *Pavona*, *Physogyra*, *Platygyra*, *Plerogyra*, *Porites* encrusting, *Porites* massive, *Porites* submassive |
| **Other/NA** | *Coeloseris*, *Coscinaraea*, *Ctenactis*, *Cycloseris*, *Euphyllia*, *Goniopora*, *Halomitra*, *Isopora*, *Oxypora*, *Pectinia*, *Pocillopora*, *Polyphillia*, *Scolymia*, *Symphyllia*, *Tubastrea*, *Tubipora* |

‘Other’ represents corals that could not be categorised into the other 4 groups.
