# Supplementary material for: Coral Reef Community Composition in the Context of Disturbance History on the Great Barrier Reef, Australia
Source: PLoS One. 2014 Jul 1;9(7):e101204. doi: 10.1371/journal.pone.0101204 (PMC4077760; doi:10.1371/journal.pone.0101204)
Supplement: Table S2 — Random factor “reef” results of the hierarchical models for a) hard coral cover, b) structural complexity and c) coral genus richness. The intercepts and 95% confidence intervals (CI) of each reef and their corresponding disturbance are presented. (DOCX) [file pone.0101204.s005.docx]

**Table S2.** Random factor “reef” results of the hierarchical models for a) hard coral cover, b) structural complexity and c) coral genus richness. The intercepts and 95% confidence intervals (CI) of each reef and their corresponding disturbance are presented.

| **Disturbance** | **Reef** | **Intercept** | **- 95% CI** | **+ 95% CI** |
| --- | --- | --- | --- | --- |
| **a) Hard coral cover** |  |  |  |  |
| Unrecovered | John Brewer | -16.08 | -19.25 | -12.90 |
| Unrecovered | Trunk | -15.75 | -18.93 | -12.57 |
| Recovered | Rib | 6.92 | 3.74 | 10.10 |
| Undisturbed | Davies | 11.64 | 8.47 | 14.82 |
| Undisturbed | Wheeler | 13.26 | 10.08 | 16.44 |
| **b) Structural complexity** |  |  |  |  |
| Unrecovered | John Brewer | -0.16 | -0.58 | 0.26 |
| Unrecovered | Trunk | -0.29 | -0.71 | 0.13 |
| Recovered | Rib | -0.02 | -0.43 | 0.40 |
| Undisturbed | Davies | 0.27 | -0.15 | 0.68 |
| Undisturbed | Wheeler | 0.20 | -0.22 | 0.62 |
| **c) Coral genus richness** |  |  |  |  |
| Unrecovered | John Brewer | -3.95 | -5.70 | -2.20 |
| Unrecovered | Trunk | -4.00 | -5.75 | -2.25 |
| Recovered | Rib | -0.96 | -2.71 | 0.79 |
| Undisturbed | Davies | 5.92 | 4.17 | 7.67 |
| Undisturbed | Wheeler | 2.99 | 1.24 | 4.74 |
