# Supplementary material for: Coral Reef Community Composition in the Context of Disturbance History on the Great Barrier Reef, Australia
Source: PLoS One. 2014 Jul 1;9(7):e101204. doi: 10.1371/journal.pone.0101204 (PMC4077760; doi:10.1371/journal.pone.0101204)
Supplement: Table S3 — Pairwise tests among reefs for PERMANOVA results on coral community composition data. (DOCX) [file pone.0101204.s006.docx]

**Table S3** Pairwise tests among reefs for PERMANOVA results on coral community composition data.

| **Groups** | **t** | **P(perm)** |
| --- | --- | --- |
| Davies, John Brewer | 8.2582 | 0.001 |
| Davies, Rib | 2.8705 | 0.001 |
| Davies, Trunk | 6.4047 | 0.001 |
| Davies, Wheeler | 1.6749 | **0.055** |
| John Brewer, Rib | 6.2534 | 0.001 |
| John Brewer, Trunk | 0.61185 | **0.793** |
| John Brewer, Wheeler | 7.3081 | 0.001 |
| Rib, Trunk | 5.2532 | 0.001 |
| Rib, Wheeler | 1.2888 | **0.196** |
| Trunk, Wheeler | 5.932 | 0.001 |

P(perm) values in bold show pairs of reefs that are not different in coral community composition.
