# Supplementary material for: Coral Reef Community Composition in the Context of Disturbance History on the Great Barrier Reef, Australia
Source: PLoS One. 2014 Jul 1;9(7):e101204. doi: 10.1371/journal.pone.0101204 (PMC4077760; doi:10.1371/journal.pone.0101204)
Supplement: Table S4 — Hierarchical model results assessing exposure and zone and their interaction with reef as a random factor for: a) competitive, b) non competitive, c) stress-tolerant, d) weedy, e) generalist, f) other coral cover (%). (DOCX) [file pone.0101204.s007.docx]

**Table S4**. Hierarchical model results assessing exposure and zone and their interaction with reef as a random factor for: a) competitive, b) non competitive, c) stress-tolerant, d) weedy, e) generalist, f) other coral cover (%).

|  | **Value** | **Std.Error** | **Df** | **t-value** | **p-value** | **Sig** |
| --- | --- | --- | --- | --- | --- | --- |
| **a) Competitive coral cover (%)** | |  |  |  |  |  |
| (Intercept) | 31.12 | 4.34 | 80 | 7.178 | 0.0000 | *** |
| zoneFlat | -14.65 | 3.35 | 80 | -4.369 | 0.0000 | *** |
| zoneSlope | -13.07 | 3.35 | 80 | -3.899 | 0.0002 | *** |
| expoSheltered | -4.15 | 3.35 | 80 | -1.238 | 0.2194 |  |
| **b) Non competitive coral cover (%)** | |  |  |  |  |  |
| (Intercept) | 7.37 | 3.28 | 80 | 2.248 | 0.0274 | * |
| zoneFlat | -1.66 | 2.40 | 80 | -0.693 | 0.4900 |  |
| zoneSlope | 14.85 | 2.40 | 80 | 6.194 | 0.0000 | *** |
| expoSheltered | 4.88 | 2.40 | 80 | 2.038 | 0.0449 | * |
| **c) Stress tolerant coral cover (%)** | |  |  |  |  |  |
| (Intercept) | 2.22 | 1.99 | 80 | 1.115 | 0.2681 |  |
| zoneFlat | 1.37 | 1.54 | 80 | 0.887 | 0.3775 |  |
| zoneSlope | 8.62 | 1.54 | 80 | 5.578 | 0.0000 | *** |
| expoSheltered | 4.63 | 1.54 | 80 | 2.994 | 0.0037 | ** |
| **d) Weedy coral cover (%)** |  |  |  |  |  |  |
| (Intercept) | 1.30 | 0.37 | 80 | 3.565 | 0.0006 | *** |
| zoneFlat | -1.25 | 0.44 | 80 | -2.837 | 0.0058 | ** |
| zoneSlope | 2.03 | 0.44 | 80 | 4.605 | 0.0000 | *** |
| expoSheltered | -0.08 | 0.44 | 80 | -0.187 | 0.8518 |  |
| **e) Generalist coral cover (%)** | |  |  |  |  |  |
| (Intercept) | 0.15 | 0.37 | 80 | 0.410 | 0.6831 |  |
| zoneFlat | -0.08 | 0.46 | 80 | -0.182 | 0.8557 |  |
| zoneSlope | 2.40 | 0.46 | 80 | 5.260 | 0.0000 | *** |
| expoSheltered | 0.37 | 0.46 | 80 | 0.804 | 0.4235 |  |
| **f) Other coral cover (%)** |  |  |  |  |  |  |
| (Intercept) | 3.70 | 0.90 | 80 | 4.123 | 0.0001 | *** |
| zoneFlat | -1.70 | 0.77 | 80 | -2.196 | 0.0310 | * |
| zoneSlope | 1.80 | 0.77 | 80 | 2.329 | 0.0224 | * |
| expoSheltered | -0.02 | 0.77 | 80 | -0.032 | 0.9749 |  |

Significance levels: ***<0.001, **<0.01, *<0.05. Crest and exposed are the intercept. The interaction of zone and exposure had a significant impact on non-competitive corals, stress tolerant corals, and generalist corals.
