# Supplementary material for: Coral Reef Community Composition in the Context of Disturbance History on the Great Barrier Reef, Australia
Source: PLoS One. 2014 Jul 1;9(7):e101204. doi: 10.1371/journal.pone.0101204 (PMC4077760; doi:10.1371/journal.pone.0101204)
Supplement: Table S5 — Random factor “reef” results of the hierarchical models for a) competitive, b) non competitive, c) stress tolerant, d) weedy, e) generalist, f) other coral cover (%). The intercepts and 95% confidence intervals (CI) of each reef and their corresponding disturbance are presented. (DOCX) [file pone.0101204.s008.docx]

**Table S5.** Random factor “reef” results of the hierarchical models for a) competitive, b) non competitive, c) stress tolerant, d) weedy, e) generalist, f) other coral cover (%). The intercepts and 95% confidence intervals (CI) of each reef and their corresponding disturbance are presented.

| **Disturbance** | **Reef** | **Intercept** | **- 95% CI** | **+ 95% CI** |
| --- | --- | --- | --- | --- |
| **a) Competitive coral cover (%)** | |  |  |  |
| Unrecovered | John Brewer | -9.31 | -11.76 | -6.85 |
| Unrecovered | Trunk | -8.68 | -11.14 | -6.23 |
| Recovered | Rib | 8.34 | 5.88 | 10.79 |
| Undisturbed | Davies | 2.70 | 0.24 | 5.15 |
| Undisturbed | Wheeler | 6.96 | 4.50 | 9.41 |
| **b) Non competitive coral cover (%)** | |  |  |  |
| Unrecovered | John Brewer | -6.19 | -8.35 | -4.03 |
| Unrecovered | Trunk | -6.04 | -8.20 | -3.89 |
| Recovered | Rib | -1.35 | -3.51 | 0.81 |
| Undisturbed | Davies | 8.26 | 6.10 | 10.42 |
| Undisturbed | Wheeler | 5.33 | 3.17 | 7.48 |
| **c) Stress tolerant coral cover (%)** | |  |  |  |
| Unrecovered | John Brewer | -3.58 | -5.24 | -1.92 |
| Unrecovered | Trunk | -3.65 | -5.31 | -1.99 |
| Recovered | Rib | -0.85 | -2.51 | 0.81 |
| Undisturbed | Davies | 4.56 | 2.90 | 6.22 |
| Undisturbed | Wheeler | 3.52 | 1.86 | 5.18 |
| **d) Weedy coral cover (%)** |  |  |  |  |
| Unrecovered | John Brewer | -0.32 | -0.88 | 0.24 |
| Unrecovered | Trunk | -0.31 | -0.87 | 0.25 |
| Recovered | Rib | -0.14 | -0.70 | 0.42 |
| Undisturbed | Davies | 0.56 | 0.00 | 1.12 |
| Undisturbed | Wheeler | 0.21 | -0.35 | 0.78 |
| **e) Generalist coral cover (%)** | |  |  |  |
| Unrecovered | John Brewer | -0.30 | -0.84 | 0.23 |
| Unrecovered | Trunk | -0.20 | -0.74 | 0.33 |
| Recovered | Rib | -0.18 | -0.72 | 0.36 |
| Undisturbed | Davies | 0.49 | -0.05 | 1.03 |
| Undisturbed | Wheeler | 0.20 | -0.34 | 0.73 |
| **f) Other coral cover (%)** |  |  |  |  |
| Unrecovered | John Brewer | -1.62 | -2.71 | -0.53 |
| Unrecovered | Trunk | -1.58 | -2.66 | -0.49 |
| Recovered | Rib | -0.03 | -1.11 | 1.06 |
| Undisturbed | Davies | 2.09 | 1.00 | 3.17 |
| Undisturbed | Wheeler | 1.14 | 0.05 | 2.23 |
